# Chemical Modification of Graphene Oxide by Nitrogenation: An X-ray Absorption and Emission Spectroscopy Study

**DOI:** 10.1038/srep42235

**Published:** 2017-02-10

**Authors:** Cheng-Hao Chuang, Sekhar C. Ray, Debarati Mazumder, Surbhi Sharma, Abhijit Ganguly, Pagona Papakonstantinou, Jau-Wern Chiou, Huang-Ming Tsai, Hung-Wei Shiu, Chia-Hao Chen, Hong-Ji Lin, Jinghua Guo, Way-Faung Pong

**Affiliations:** 1 Department of Physics, Tamkang University, Tamsui 251, New Taipei City, Taiwan; 2 Department of Physics, University of South Africa, Florida Science Campus-1710, Johannesburg, South Africa; 3 Engineering Research Institute, School of Engineering, Ulster University, BT37 0QB, Newtownabbey, UK; 4 Department of Applied Physics, National University of Kaohsiung, Kaohsiung 811, Taiwan; 5 National Synchrotron Radiation Research Center, Hsinchu 300, Taiwan; 6 Advanced Light Source, Lawrence Berkeley National Laboratory, Berkeley, California 94720, USA; 7 Department of Chemistry and Biochemistry, University of California, Santa Cruz, California 95064, USA

## Abstract

Nitrogen-doped graphene oxides (GO:N_x_) were synthesized by a partial reduction of graphene oxide (GO) using urea [CO(NH_2_)_2_]. Their electronic/bonding structures were investigated using X-ray absorption near-edge structure (XANES), valence-band photoemission spectroscopy (VB-PES), X-ray emission spectroscopy (XES) and resonant inelastic X-ray scattering (RIXS). During GO:N_x_ synthesis, different nitrogen-bonding species, such as pyrrolic/graphitic-nitrogen, were formed by replacing of oxygen-containing functional groups. At lower N-content (2.7 at%), pyrrolic-N, owing to surface and subsurface diffusion of C, N and NH is deduced from various X-ray spectroscopies. In contrast, at higher N-content (5.0 at%) graphitic nitrogen was formed in which each N-atom trigonally bonds to three distinct *sp*^2^-hybridized carbons with substitution of the N-atoms for C atoms in the graphite layer. Upon nitrogen substitution, the total density of state close to Fermi level is increased to raise the valence-band maximum, as revealed by VB-PES spectra, indicating an electron donation from nitrogen, molecular bonding C/N/O coordination or/and lattice structure reorganization in GO:N_x_. The well-ordered chemical environments induced by nitrogen dopant are revealed by XANES and RIXS measurements.

The chemical toughness of a single-layered graphene sheet is important for electron transport and electro-catalytic reactions, and especially for interior (crystalline structure) and exterior (nano-composite) modifications of graphene-like structures^1^,^2^. The semiconducting properties that support nano-electronics^3^ and light-energy harvesting applications^4^,^5^ require opening of the energy gap of graphene. Chemical doping^6^, substrate induction^7^, and crystallographic orientation^8^ can all affect the host state, charge transfer, and changes in the electronic density of states (DOSs)^9^. Doping with boron or nitrogen atoms as the electron-acceptors or donors through carbon substitution, respectively, is commonly conducted to control the Fermi level (*E*_f_) and metal-semiconductor transition^10^,^11^,^12^. Decoration of the graphene structure with hydroxyl and epoxy groups, to form chemically derived graphene oxide (GO) and reduced graphene oxide (r-GO) in a controllable oxidation and reduction step^12^, favors large-volume synthesis and chemical solubility in a variety of solvents. In fact, the chemical doping of graphene can lead to the formation of external covalent bonds in contrast to the six-membered structure and prevent the conjugation of π-states. The removal/substitution of carbon atoms, topological defects/vacancies, structural disorder, molecular absorption and symmetry breaking have been demonstrated to result in the formation of localized states and electron/hole states in the electronic structures of GO and r-GO^13^,^14^. Chemical doping affects the surface of GO and is also involved in dislocating the lattice of graphene^15^. Therefore, the mechanisms of the changing gapless property of graphene from dopant-induced perturbation and defect/vacancy-induced rearrangement need to be understood in order to develop energy storage and environmental applications^16^,^17^.

Among the various processes for synthesizing, the Hummers method^18^ is commonly used to extract individual graphene sheets that comprise serveal oxidation groups (i.e. C-OH, -COOH, C-O-C and C = O bond). In this process, highly oriented pyrolytic graphite (HOPG) is exfoliated and then dispersed into an aqueous solution. Various methods for controllably de-oxidizing functional groups, such as reduction with hydrazine^19^, sodium borohydride^20^, hydrothermal treatment^21^ and thermal annealing^22^, are used to inactivate the chemical connection between epoxide/hydroxyl groups in the GO-basal network. However, these processes desorb the covalently bonded oxygen and have two important effects, namely mixing the metallic *sp*^2^ domain and the insulating *sp*^3^ matrix, and forming structural defects/vacancies in the structural configuration^23^. Instead of intensively breaking oxygen molecules by these reduction processes and relieving unpaired electrons, nitrogen-associated replacement can be used to decompose the oxygen-containing functional groups because nitrogen has a similar atomic radius to oxygen and the chemical hybridization^24^. Li *et al*.^25^ developed a chemical method to obtain N-doped GO through thermal annealing of GO in ammonia at various temperatures; where they found that the content of doped nitrogen achieved by the replacement of oxygen-containing functional groups is proportional to the increase in temperature. The electrical conductivity measurement revealed that the Dirac point is at a negative gate voltage because nitrogen is an *n*-type electron dopant^26^. Since nitrogen and oxygen co-exist in the graphene sheet, along with structural defects, the effects of chemical doping and/or nitrogenation in GO warrant intensive study. Among the various techniques for studying element-specific absorption, the X-ray absorption near-edge structure (XANES) spectroscopy can be used to detect the unoccupied states in the conduction-band^24^ and X-ray emission spectroscopy (XES) is used to probe the occupied states in the valence-band of the material^27^. XANES/XES is element-specific and site-specific, making it a powerful tool for elucidating detailed electronic properties of materials. Owing to the momentum conservation between the excited electron in the conduction band and decayed valence-band hole, resonant inelastic X-ray scattering (RIXS) is used to probe the symmetry points along the band structures relying on its doping, reduction and lattice effect^26^. The graphene-related and GO/r-GO materials have been found to demonstrate novel electronic and magnetic properties resulting from different electronic structure and bonding environment, offering the working mechanism for the future use^21^,^28^,^29^,^30^,^31^.

In the present study, GO was used as a starting material to elucidate the mechanism of the nitrogen substitution by replacing the oxygen-related bonds, and to verify the modification of electronic/bonding structures and changes in the binding energy of GO:N_x_ by the partial reduction of GO with urea using household microwave. This process is simple and differs from several other reported approaches^19^,^25^,^26^,^32^,^33^. In this approach, the chemical environments in GO:N_x_ are monitored by studing the C, N, and O at various concentrations of nitrogen; the effect on band gap opening (or energy separation) of the activation of nitrogen and oxygen atoms in the graphene-basal plane network is also examined. For this purpose, various forms of spectroscopy, including Raman spectroscopy, X-ray photoelectron spectroscopy (XPS), valence-band photoemission spectroscopy (VB-PES), and combined XANES/XES and RIXS techniques were used.

## Results and Discussion

**Figure 1:**
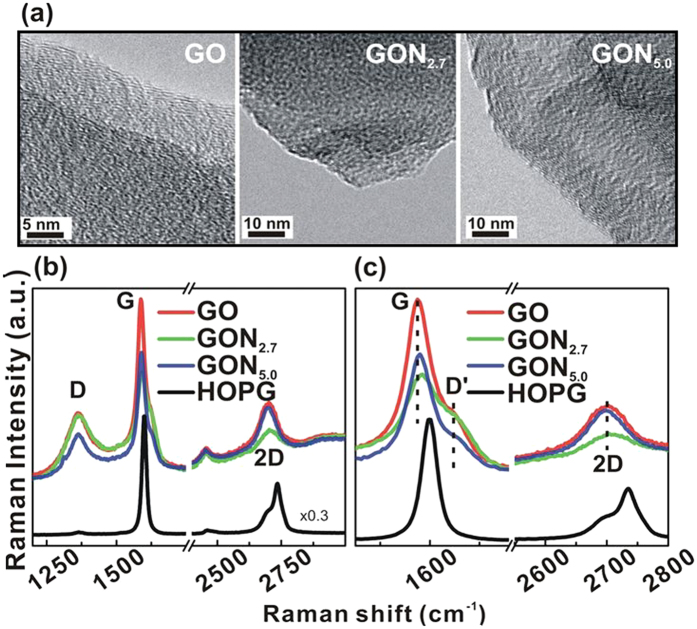
(**a**) TEM images of (**a**) GO and GO:N_x_ (x = 2.7 and 5.0) and (**b**) Raman spectra of GO, GO:N_x_ (x = 2.7 and 5.0) and HOPG.

Initially, GO is synthesized herein by the Hummers method^18^, and then mixed with urea to synthesize GO:N_x_ in a household microwave at 700 W for various durations. The surface morphologies of GO and GO:N_x_ are investigated using transmission electron microscopic (TEM) images, which show the uniform stacking of multi-layered GO/GO:N_x_, as presented in Fig. 1(a). The elements in GO/GO:N_x_ (at% of C, O and N) are quantified using TEM-energy dispersive spectroscopy (EDS). The TEM-EDS study revealed that the oxygen content gradually decreases in the order from 32.5 (GO) → 26.2 (GO:N_2.7_) → 19.3 at% (GO:N_5.0_) as the carbon (nitrogen) content increases in the order from 67.5(0) → 71.1(2.7) → 75.7(5.0) at% in GO and GO:N_x_. These quantitative results imply that treatment with nitrogen induces oxygen reduction and carbon purification, facilitating nitrogen substitution in GO:N_x_ (x = 2.7 & 5.0 at %). The microstructural properties of GO and GO:N_x_ are studied by Raman spectroscopy, as presented in Fig. 1(b). The Raman spectrum of the pristine GO exhibits three characteristic features: the *D* band at ~1362 cm^−1^, the *G* band at ~1587 cm^−1^ and the 2*D* band at ~2702 cm^−1^. The *G* band and *D* band yield information about the lattice structure and about graphene-related *E*_2g_ phonon and defect-resolved *A*_1g_ symmetry, respectively^34^. The intensities of all features (*D, G* and 2*D*) of GO:N_x_ significantly decrease. In GO and GO:N_x_, the splitting of *G*-peaks in two components (*G* at ~1587 and D′ at ~1629 cm^−1^) is attributed to the mixed signal from the disordered graphene lattice upon nitrogen doping^35^. The peak at ~2455 cm^−1^ and a very wide peak at ~2920 cm^−1^ arise owing to a combination of (*D* + *D*′) bands, which is seen in defect-activated graphene oxides^29^. The feature of *D* is also defect-activated in an inter-valley double resonance process and its intensity is a convenient measure of the degree of disorder^29^. The decrease in the intensity of *D* band (1362 ± 1 cm^−1^) and the presence of a new band (*D*′) at ~1629 (±1) cm^−1^ can be attributed to the attachment of nitrogen on the top layer or the inter-layer of GO, compared with the well-ordered lattice of HOPG. Upon nitrogenation, the 2*D* band of GO:N_x_ is reduced and slightly shifted, and the I_2*D*_/I_*G*_ ratio changes, as displayed in Fig. 1(b) and (c). The intensity ratio I_*D*_/I_*G*_ decreases, and the I_*D*_ and I_*D*′_ ratio increase with an increase in N at% doping. The second-order *D* peak (2*D*) at ~2702 cm^−1^ is associated with the two-phonon mode, in which the phonons have opposite momentum, close to the *K* point of the Brillouin zone. Various N at% in GO:N_x_ causes the shape and intensity of the peak to differ significantly from that of the highly ordered HOPG. The (I_2*D*_/I_*G*_) ratios of both GO and GO:N_x_ are less than one, which is indicative of the formation of tri- or multi-layered GO/GO:N_x_, as discussed in our earlier study^29^. Liu *et al*.^36^ probed layer-dependent GO using Raman scattering and observed that oxidation increases the frequencies of the *D, G* and 2*D* modes of tri-/multi-layer graphene. Liu *et al*. concluded that oxidation by O_2_ creates strong ‘hole’ doping (*p*-type) in the graphene surface. In the present study, GO is converted into GO:N_x_ by the removal of oxygen-containing functional groups in a de-oxidation process, so the peaks are shifted downward in frequency closed to HOPG. Also, nitrogenation shifts the *G*-band from 1587 cm^−1^ (GO) to 1592 (1589) cm^−1^ in GO:N_2.7_ (GO:N_5.0_) by changing the charge density^37^. This observation clearly indicates the doping of nitrogen along with the simultaneous removal of oxygen-containing functional groups and intrinsic *n*-type conduction.

**Figure 2:**
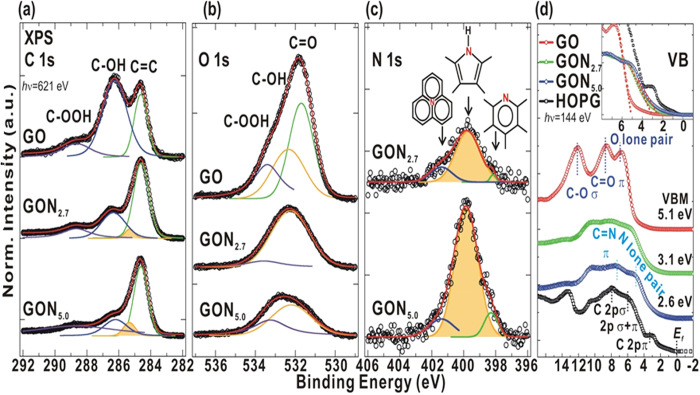
(**a**) C 1 *s*, (**b**) O 1 *s* and (**c**) N 1 *s* XPS of GO and GO:N_x_ (x = 2.7 and 5.0), respectively. Open circles represent measured spectra, and red solid curves represent fitted results. Each solid curve is fitted using Voigt-shaped function following background subtraction. (**d**) Valence-band of GO, GO:N_x_ (x = 2.7 and 5.0) and HOPG. Inset (**d**) magnifies the region of VBM or *E*_f_ of GO, GO:N_x_ (x = 2.7 and 5.0) and HOPG.

Figure 2(a–c) show the N, C and O 1 *s* core-level XPS of the individual chemical binding environment in GO and GO:N_x_. All measured spectra are plotted as black circles and their fitted curves are in different colors; these are obtained by the peak-fitting routine for a minimum of the difference function (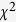) as for the purely statistical noise. The components of the fitting procut are described by the parameters, Shirley/linear background and individual Voigt-function peak (position constrained within 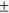
0.1 eV). In Fig. 2(a), the wide asymmetric C 1 *s* complex band of GO has two maxima and one minimum features which arise gradually as one strong and two weak features, owing to the formation of GO:N_x_. All C 1 *s* core-level XPS spectra are well-fitted by four features using the minimum 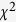 search^38^,^39^. These features are attributed to C = C (plum) at ~284.6 eV, C-OH (green) at ~286.3 eV, C-OOH (blue) at ~288.7 eV and C-N bonds (filled yellow) at ~285.4 eV^40^,^41^. The number of oxygen-containing functional groups in the GO:N_x_ structure gradually decrese as N-doping increases. This change in the reduction of the oxygen-containing functional groups is observed in the O 1 *s* XPS spectra in Fig. 2(b). Figure 2(b) presents a wide asymmetric O 1 *s* core-level XPS spectrum complex band of GO, that is comprised of various oxygen-related bonds, and is converted to less intense features in GO:N_x_. The fitting yields three features, which are C = O (shaded plum) at ~531.6 eV, C-OH (green) at ~532.4 eV and C-OOH (blue) at ~533.2 eV^42^. In Fig. 2(c), the intensity of N 1 *s* XPS spectrum increases with the nitrogen concentration. The fitting of N 1 *s* spectra yields three features, which are centered at ~398.2, ~399.8, and 401.4 eV, and are assigned the pyridinic N at the edge of six-membered ring (plum), the pyrrolic N structure (yellow), and the quaternary N structure (green), respectively^25^. The peak at ~401.4 eV becomes more intense as the nitrogen concentration increases^25^, indicating the formation of a more graphitic structure. Overall, the above results reveal that the intensities of oxygen-containing functional groups in C 1 *s*/O 1 *s* XPS spectra decrease and the intensity of the N 1 *s* XPS spectra increases with the increase in nitrogen content in the GO:N_x_. These further confirmed the removal of oxygen-containing functional groups (C = O and C-OH) and an increase in the numbers of nitrogen-related pyrrolic, pyridinic, quaternary-N groups in the GO:N_x_.

The occupied valence DOSs of GO and GO:N_x_; the total DOSs below valence-band maximum (VBM) or *E*_f_ was obtained by using the VB-PES of GO, GO:N_x_ and HOPG for a reference spectra, as presented in Fig. 2(d). In this figure, HOPG has four distinct hybridized states, which are C 2*p*-π at ~3.0 eV, C 2*p* σ + π at 6.0 eV, C 2*p*-σ at 8.0 eV and C 2*s*-σ at 10.0 eV^43^. In pristine GO, the features at ~12.1, 8.6, and 6.7 eV are attributed to the σ and π bonding of C = O, and the O lone pair^41^ and overlaps C 2*p*-(σ + π) and C 2*p*-σ aromatic features of HOPG. The prominent nitrogen-associated bands, C = N at ~7.3 eV and the N lone pair at ~5.0 eV, are clearly observed in GO:N_x_ (x = 5.0 at%)^44^,^45^. These two features are related to the *p*-σ and *p*-π contributions to the DOSs, respectively, as also observed in our earlier study of *a*-CN_x_ thin films^46^. The VBM was determined from the extrapolation of the threshold-edge of the VB-PES spectra of HOPG and GO:N_x_ to the baseline, shown in the upper inset in Fig. 2(d). The VBM position as 5.1 eV (GO) → 3.1 eV (GON_2.7_) → 2.6 eV (GON_5.0_) → 1.8 eV (HOPG), indicating an increase of DOSs at/near VBM or *E*_f_ with an increase in nitrogen concentration in GO:N_x_^9^. This effect could be due to the formation of the N-lone-pair electrons, π-electrons of C-N bonds and σ-electrons of C-N bonds as nitrogen content increases^9^; however, an open question for VBM shift should be related to the lattice rearrangement and oxygen/nitrogen bonding groups, decreasing work function of GO:N_x_^47^. This result is consistent with the electrical conductivity measurements, which reveal that the Dirac point is at a negative gate voltage owing to nitrogen as *n*-type electron doping in nitrogen-doped graphene^48^, confirming the change in chemical composition and bonding modification with the active involvement of nitrogen in the network of oxygen-containing functional groups and the carbon matrix structure.

**Figure 3:**
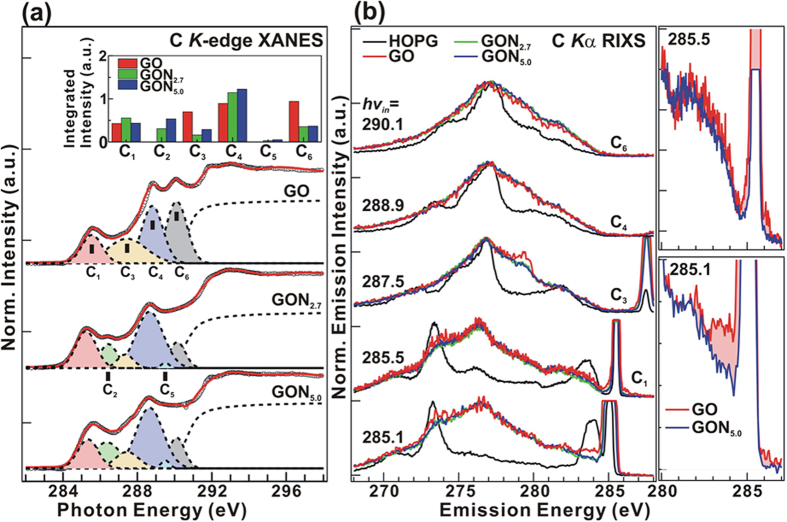
(**a**) C 1 *s* XANES and de-convoluted features. The inset is the intensity bar diagram of C 1 *s* XANES features of GO and GO:N_x_ (x = 2.7 and 5.0), obtained by fitting. (**b**) C *K*_α_ RIXS spectra of GO and GO:N_x_ (x = 2.7 and 5.0).

Figure 3(a) displays the normalized and fitting C *K*-edge XANES spectra of GO and GO:N_x_. De-oxidation and nitrogenation significantly change the spectral features and line-shapes of GO. The spectra of GO and GO:N_x_ are fitted into four features C_1_, C_3_, C_4_, and C_6_ and six features C_1_-C_6_, respectively, as presented in Fig. 3(a). These features are assigned to C_1_ at ~285.5 eV (C = C bond)^49^, C_2_ at ~286.4 eV [1 *s* → π* (*e*_2u_) transition similar to that of pyridine (C = N)]^46^,^50^,^51^, C_3_ at ~287.4 eV (π* C-H/C-OH)^46^,^52^, C_4_ at ~288.7 eV (π* C-OOH/C = O)^46^,^52^, C_5_ at ~289.5 eV (C-N bond with the carbonyl functionality nearby)^46^,^50^,^51^ and C_6_ at ~290.1 (likely C-H bond)^53^. The feature of GO observed at ~285.2 eV (C_1_) is the C = C bonds in the six-membered ring, which is shifted to a higher energy of ~285.5 eV. Its intensity increases upon chemical decoration by nitrogen-related functional groups^49^ to form GO:N_x_ owing to de-oxidation in the presence of urea. During de-oxidation, nitrogen in GO:N_x_ structure is bonded to carbon (C_2_: 286.4 eV and C_5_: 289.5 eV)^46^,^50^,^51^ by the changing the numbers of π* C-OH (C_3_: 287.4 eV), π* C-OOH/C = O (C_4_: 288.7 eV)^46^,^52^ and C-H bonds (C_6_: 290.1 eV)^53^. The intensities of all these features vary upon de-oxidation/nitrogenation, as clearly shown in the intensity bar diagram in the inset of Fig. 3(a), consistent with the above XPS results. The increase in the number of *sp*^2^ clusters in the form of pyridine-like N-C and graphite-N-like (C_2_ and C_5_) bonds and the resonant energy and charge transfer^54^ from the N and O atoms to the *sp*^2^ clusters involve de-oxidation, nitrogenation, change in structural-ordered and, therefore, changes in the chemical, composition, electronic structure and bonding coordination of GO:N_x_. However, to verify directly the increase in the number of substitution-induced localized electronic states in GO:N_x_ upon nitrogenation, a detailed analysis of the DOSs in the region of π or in the π-π* gap was conducted using RIXS^21^. This technique is a powerful method for investigating the incident coherent excitation (*hv*_*in*_) and emitted-energy dependence that is element specific and allows sensitive probe of the symmetry points of the Brillouin zone^55^. Figure 3(b) displays the C *K*_α_ RIXS spectra of GO, GO:N_x_ and HOPG, obtained by excitation with various *hv*_*in*_. All of the emission spectra were obtained within the same period and were normalized to yield an intensity of unity for the maximum inelastic emission peak in each spectrum. According to RIXS theory, the total energy and momentum must be conserved in resonant inelastic scattering in absorption-emission processes^56^,^57^. The *hv*_*in*_-dependent emission features that are identified from the HOPG results from a transition at a well-orientated crystal momentum. Figure 3(b) shows that the C *K*_α_ RIXS spectra of GO, GO:N_x_ and HOPG depend strongly on the excitation energy, and this dependence is believed to arise from the non-resonant inelastic scattering, related to the similarity in the band structures of GO and GO:N_x_. The GO/GO:N_x_ spectra exhibit different line-shapes and emission features (σ and π) that appear slightly dispersive, particularly within the range 280–285 eV, differing clearly from the HOPG spectrum. The π emission derives from transitions from *p*_z_ states that are oriented perpendicular to the graphitic plane. Emission from the σ bands is much more isotropic because these bands are derived partially from the *p*_x_ and *p*_y_ states that are parallel to the surface. The emitted photon energies (*h*ν_*out*_) vary with *hv*_*in*_ from 285.1 to 290.1 eV. The *h*ν_in_-dependent emission features arise from transitions from states with a well-defined crystal momentum^56^,^57^. The σ orbital at ~273.4 eV and π orbital at ~284.0 eV of HOPG are shrunk by the *k*-conserving partial DOSs in the well-defined crystal momentum as a function of the *hv*_*in*_ energy^58^. The emission spectra of GO:N_x_ are insensitive to on-site elastic scattering and become broader in different *hv*_*in*_-energies as compared to that of HOPG spectrum, which is characteristic of structural disorder effects as additional oxygen and nitrogen atoms are dispersed over the band structures^59^. The π-features in the C *K*_α_ RIXS spectra of the GO/GO:N_5.0_ change with incident photon energy from 285.5 to 285.1 eV, as presented in right-hand panels of Fig. 3(b), in a very different way from those of HOPG. This is because the excitation energies at the various absorption edges of carbons that are bonded to oxygen and/or oxygen-containing functional groups generate more resonant π-features in GO/GO:N_5.0_ than in HOPG. The DOSs of the pristine GO is higher than that of GO:N_x_ with a shoulder just above *E*_f_ and is observed more prominently at *hv*_*in*_ = 285.5 eV [inset in Fig. 3(b)]. This DOS is highly sensitive to the electronic DOS close to *E*_f;_ as observed by Zhong *et al*.^60^ in the C *K*_α_ emission spectra of single wall nanotubes. This feature of GO at ~283.3/282.6 eV (*hv*_*in*_ = 285.1/285.5 eV) is not observed in the spectrum of the GO:N_x_ structure because the localizations of carbon core-hole orbitals changed upon the reconstruction of the nitrogen-doped states^61^. This spectral change is caused by mixing of the π-σ range upon de-oxidation/nitrogenation and the contribution of the C-OH-like feature, C_3_-, in the C *K*-edge XANES spectra. This revealed that the removal of oxygen and/or oxygen-containing functional groups from the GO lattice and induced nitrogen-related states, changed the DOSs in the π-region and/or in the gap between π and π* states.

**Figure 4:**
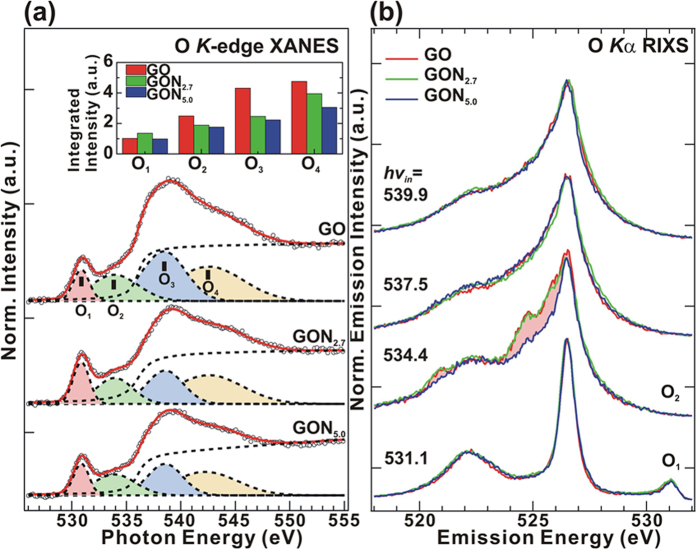
(**a**) O 1 *s* XANES and de-convoluted features. The inset is the intensity bar diagram of O 1 *s* XANES features of GO and GO:N_x_ (x = 2.7 and 5.0), obtained by fitting. (**b**) O *K*_α_ RIXS spectra of GO and GO:N_x_ (x = 2.7 and 5.0).

Figure 4(a) presents the normalized O *K*-edge XANES spectra of GO and GO:N_x_. All spectra are fitted into four features (O_1_-O_4_), and their intensities are shown in Fig. 4(a). A sharp 1 *s* → π* C = O (O_1_) resonance at ~530.9 eV and intense 1 *s* → σ* C-OH resonance at ~538.5 eV (O_3_) with two shoulders π* C-O-C/C-OH at ~533.9 eV (O_2_) and σ* C-O/C = O ~542.4 eV (O_4_) are observed^52^,^62^. The intensities of these features decreased upon de-oxidation/nitrogenation, as clearly revealed in the intensity bar diagram in the inset of Fig. 4(a). These results confirmed the removal of different oxygen-containing functional groups (C = O and C-OH) from the GO surface and the inclusion of nitrogen that is bonded with carbon and oxygen (pyrrolic, pyridinic, quaternary-N groups) in GO:N_x_ as the nitrogen content increases in the GO matrix. To investigate the potential removal of oxygen-containing functional groups, the O *K*_α_ RIXS of GO and GO:N_x_ with *hv*_*in*_ of 531.1–539.9 eV are used, as shown in Fig. 4(b). All spectra exhibit an intense feature at ~526.5 eV with a shoulder at ~522.1 eV at various *hv*_*in*_ energies, to which π → 1 *s* and σ → 1 *s* in the valence band contribute. A strong resonant contribution is observed at *hv*_*in*_ = 534.4 eV (O_2_); the intensity of the π at 524.0–525.5 eV and the σ electron at 520.9 eV decreased as nitrogen concentration increased^63^, indicating the reduction of the C-O-C/C-OH bond in the valence band. The emission width of the π-derived state shrunk at *hv*_*in*_ = 539.9 eV owing to a loss of oxygen-containing bonds as the concentration of N increased. The RIXS profile measured at *hv*_*in*_ = ~537.5 and 539.9 eV reflects insensitive in the π- and σ-derived states, which may be a result of the incoherent yield and fluorescent superposition effect^64^.

**Figure 5:**
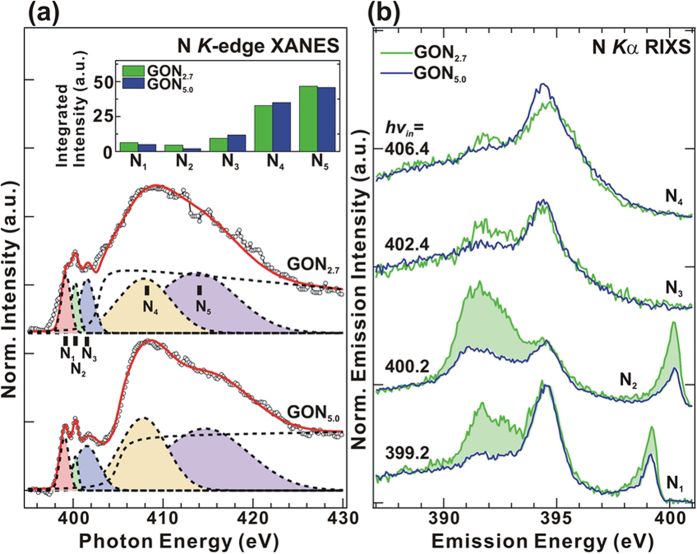
N 1 *s* (**a**) XANES and de-convoluted features, The inset is the intensity bar diagram of N 1 *s* XANES features of GO:N_x_ (x = 2.7 and 5.0), obtained by fitting. (**b**) N *K*_α_ RIXS spectra of GO:N_x_ (x = 2.7 and 5.0).

Figure 5(a) displays the normalized N *K*-edge XANES spectra of GO:N_x_, which provide evidence concerning the local electronic states that are bound to the nitrogen sites. The spectra are fitted into five features, whose intensity bars are presented in the inset of Fig. 5(b). In the π* region, a strong feature (N_2_) at ~400.2 eV is assigned to the -C≡N triplet in GO:N_2.7_^51^. Close to this feature, two shoulders at ~399.2 eV (N_1_) and at ~401.5 eV (N_3_) are attributed to the pyridinic/pyrrolic N-related group with two carbon neighbors in six/five-membered ring and the nitrogen-induced substitution in graphite and/or N-O bonding^46^,^51^,^65^. In the σ*-region, the 1 *s → *σ* transition at ~406.4 eV (N_4_) is associated with the superposition of graphite-like and pyridine-like nitrogen structures^46^ and/or photoionization of N 1 *s* electrons^66^, whereas the broad feature at ~415 eV (N_5_) indicates the σ* state of pyridinic structure on the surface of GO:N_x_^67^. The intensity of the N_1_/N_2_ feature slightly decreases; whereas those of features N_3_ and N_4_ increases as the nitrogen concentration (5 at.%) increases, indicating the decrease of non-graphene-like structure (pyridinic/pyrrolic N and -C≡N triplet) and increase of graphene-like structure (N-substitutional site) [(Fig. 5(a)]. As displayed in Fig. 5(b), N *K*_α_ RIXS emission was used to probe with the lower (2.7 at.%) and higher (5 at.%) nitrogen concentrations and to provide partial DOS information with π-bonding electrons at ~394.5 eV and σ-bonding electrons at ~391.7 eV. Interestingly, the dispersive features between σ and π orbitals and their line shapes differ greatly from each other. When the resonant (*hv*_*in*_) energies are 399.2 and 400.2 eV, the RIXS profiles at low N content (2.7 at.%) include more intense features at the π-state (~391.7 eV) and the σ-state (~392.7 eV) than at a higher nitrogen content (5 at.%), owing to the pyridine (N_1_) and -C≡N triplet bond (N_2_) in the GO:N_x_. During de-oxidation/nitrogenation process, various N-O bonds are expected to be formed in the GO network, and the oxygen/oxygen-containing functional groups in GO:N_x_ (x = 5.0 at%) are strongly reduced, as revealed by the O *K*_α_ RIXS emission spectra in Fig. 4(a). At the resonant energy *hv*_*in*_ = 406.4 eV, the RIXS profiles with the high N-content (5.0 at.%) show more intense features associated with π-bonding electrons at ~394.5 eV than that with the lower N-content (2.7 at.%), indicating the formation of a pyridine-like N-bonding structure, which was also observed by Hellgren & Guo *et al*.^51^ in carbon nitride films and is consistent with the appearance of the N lone-pair in VB-PES, as presented in Fig. 2(d). In the pyridine-like N-bonding structure, nitrogen not only forms a hexagonal ring structure, but is substituted for one carbon atom in the hexagonal ring, accordingly, the percentage of carbon-bonded nitrogen bonds must increase as the nitrogen concentration in the structure increases. Therefore, the atomic fraction of nitrogen in a locally ordered C-N structure differs from that of a disordered structure.

**Figure 6:**
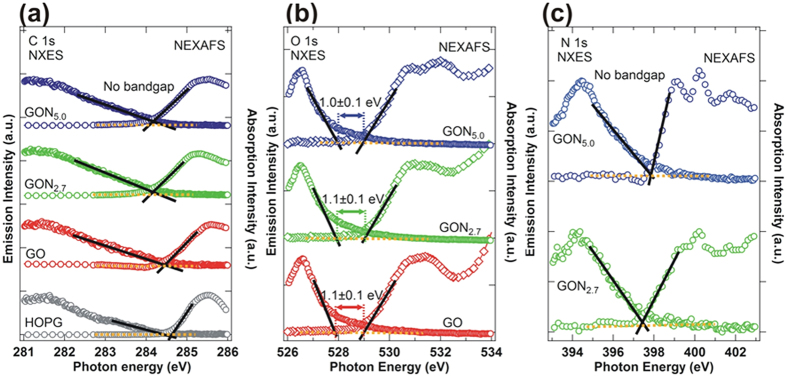
(**a**) Normalized C *K*-edge XANES and *K*_α_ XES spectra of GO and GO:N_x_ and HOPG. (**b**) Normalized O *K*-edge XANES and *K*_α_ XES spectra of GO and GO:N_x_. (**c**) Normalized N *K*-edge XANES and *K*_α_ XES spectra of GO:N_x_ (x = 2.7 and 5.0).

Figure 6 displays C, O and N *K*-edge XANES and *K*_α_ XES spectra of GO and GO:N_x_, to obtain the band gap [or energy separation between the conduction-band maximum (CBM) and VBM] of GO and GO:N_x_. Details of the derivation of energy separation in the GO from C and O *K*-edge XANES and *K*_α_ XES spectra measurements can be found elsewhere^21^. According to these spectra, the overlapping π and π* states in GO/GO:N_x_ are similar in C and N *K*-edge XANES, and *K*_α_ XES spectra exhibit no energy gap/separation, similar to that of HOPG, as displayed in Fig. 6(a) and (b). However, the O *K*-edge XANES and *K*_α_ XES spectra in Fig. 6(b) show that the energy separation between CBM and VBM is close to 1.1(±0.1) eV for GO:N_x_. This result clearly indicates that both oxygen and nitrogen are responsible for the change in the electronic structures at/near *E*_f_ in the GO:N_x_. Although the combination of individual XES spectra (as partial DOS) is equal to VB-PES spectra (as total DOS), the dipole cross-section dependence between X-ray polarization and molecular orientation during absorption and de-excitation process is the key to observe the difference between XES and VB-PES. Since the off-resonant (XES) probing is the integrated effect of whole functional group, the on-resonant (RIXS) probing intensifies the spectral weight of discrete ones with the dipole symmetry selection for electronic structure mapping. It could be related to the molecular orientation of functional groups modulated with the nitrogen concentration in GO:N_x_, revealing DOSs at/near VBM or *E*_f_ (VB-PES result) increases with the decrease of work function^47^. Souto *et al*.^9^ calculated the DOSs of N-substituted graphite clusters and found that as the N concentration in the cluster increases, the π and σ bonds are pushed to higher binding energies and new features close to *E*_f_ are formed because *sp*^2^-N in a planar graphite structure contributes two electrons to the π-system. Souto *et al*. further argued that, as the N content increases, the system adopts a three-dimensional *sp*^3^-structure, in which the N π-electrons become nonbonding lone-pairs as other electrons occupy the π orbitals of C-N bonds and the σ orbitals of C-N bonds at cluster boundaries. These results are consistent with this study, as the VBM or *E*_f_ in VB-PES spectra changes from 5.1 eV (GO) to 2.6 eV (GO:N_5.0_), indicating that DOSs at/near VBM or *E*_f_ increases with nitrogen concentration and binding environment^9^. It further supports the band gap (or energy separation) behavior that was obtained from Fig. 6 and is consistent with the results reported by King *et al*.^68^ for amorphous hexagonal boron nitride films with/without silicon doping.

In summary, different nitrogen-bonded carbon/oxygen-containing functional groups are formed by the partial removal of oxygen-containing functional groups from the GO surface through simultaneous de-oxidation/nitrogenation in the presence of urea. The dependence of the electronic and bonding properties to the N species and its concentration was extensively elucidated. The number of oxygen-containing functional groups (C-OH and C-OOH) gradually decreases from GO upon the chemical reaction of nitrogen atoms, yielding distinct features associated with the aromatic C = C state and various N-bonded carbon states, such as nitrile, pyridine-like, and graphitic-like structures in the GO:N_x_ matrix. Specific chemical environments of nitrogen, as in a disordered graphene-sheet give rise to multiple states in the valence-band structures owing to the *hv*_*in*_-dependent at C *K*_α_ RIXS. The N *K*_α_ RIXS features are quite sensitive to non-resonant *hv*_*in*_, reflecting an ordered structure at nitrogen sites, which highlights the complexity and disorder in GO:N_x_. The VBM or *E*_f_ in the VB-PES spectra changes considerably from 5.1 to 2.6 eV upon de-oxidation/nitrogenation, suggesting the preferred metallic behavior of GO:N_x_. Resonant and non-resoant emission result provides an alternative means to determine element-specific binding and electronic structure between C, O, and N sites, indicative of work function decreasing and VBM increasing. The advantage of using different X-ray spectroscopic methods could trace the electronic and magnetic origin (in particular for the element-specific proof) to the lattice environment and chemical functional groups in heterogeneous GO:N_x_ materials. This study finds that the electronic and structural properties of GO/GO:N_x_ can be tailored by de-oxidation/nitrogenation in the presence of urea, rendering GO/GO:N_x_ attractive for use in the fabrication of junction devices and field effect transistors for semiconducting and optoelectronic devices.

## Methods

### Preparation of GO and GO:N_x_

Initially, GO is synthesized by the Hummers method^18^. 50 mL of aqueous solution of GO (0.08 g) was prepared by ultra-sonicating for 50–60 minutes. 0.08 g of urea was then added and sonicated for another 5 minutes. The mixture was then put in a standard household microwave (2450 MHz, 700 W) and heated for 100, and 400 s at maximum output power. The solution was cooled to room temperature and centrifuged at 3000 rpm for 30- minutes. The clear supernatant obtained after centrifuge was separated and the brown/black precipitate were further washed in deionised water a few times by centrifuging at 3000 rpm for 30 minutes. The final slurry were left to dry in the vacuum oven at 50 °C overnight to result in GO with 2.7 at% nitrogen (GO:N_2.7_) and GO with 5 at% nitrogen (GO:N_5.0_) as confirmed by XPS measurements.

### Characterization

XANES and XES/RIXS were performed at the undulator of beamline 8.0.1 at the Advanced Light Source (ALS), Lawrence Berkeley National Laboratory. The absorption spectra of C, O, N *K*-edge are recorded in total electron yield and fluorescence yield modes at 300 K and normalized by the current of the Au grid upstream of the beamline. The energy resolution of the absorption spectra is approximately 0.10 eV for C *K*-edge, 0.30 eV for O *K*-edge, 0.25 eV for N *K*-edge XANES measurements. The XES/RIXS spectra are separately calibrated using the elastic features of HOPG, the O site of TiO_2_, and the N site of BN. Core-level XPS is performed at *E*_*hv*_ = 621 eV with an energy resolution of ~0.08 eV, and VB-PES is measured at a photon energy of 144 eV, using Beamline-09A1 at the National Synchrotron Radiation Research Center (NSRRC), Taiwan. Raman spectroscopy was conducted using an ISA LabRam system that was equipped with a 632.8 nm He-Ne laser with a spot size of approximately 2–3 mm, yielding a spectral resolution of better than 2 cm^−1^. Due care was taken to minimize heating of sample by using a low laser power of less than 2 mW to minimize desorption and/or oxidation by the laser-induced heating of de-oxidized GO. The surface morphologies of GO and GO:N_x_, were studied using TEM images. Elementals were quantified using TEM-EDS and XPS study.

## Additional Information

**How to cite this article**: Chuang, C.-H. *et al*. Chemical Modification of Graphene Oxide by Nitrogenation: An X-ray Absorption and Emission Spectroscopy Study. *Sci. Rep.* **7**, 42235; doi: 10.1038/srep42235 (2017).

**Publisher's note:** Springer Nature remains neutral with regard to jurisdictional claims in published maps and institutional affiliations.
